# Antimicrobial Activity of Tulsi (*Ocimum tenuiflorum*) Essential Oil and Their Major Constituents against Three Species of Bacteria

**DOI:** 10.3389/fmicb.2016.00681

**Published:** 2016-05-17

**Authors:** Hanaa A. Yamani, Edwin C. Pang, Nitin Mantri, Margaret A. Deighton

**Affiliations:** ^1^School of Science, Royal Melbourne Institute of Technology UniversityMelbourne, VIC, Australia; ^2^Biology, Section Microbiology, School of Applied Sciences, King Abdulaziz UniversityJeddah, Saudi Arabia

**Keywords:** Tulsi (*Ocimum tenuiflorum*), essential oil, antimicrobial activity, headspace–solid phase microextraction, gas chromatography–mass spectrometry

## Abstract

In recent years scientists worldwide have realized that the effective life span of any antimicrobial agent is limited, due to increasing development of resistance by microorganisms. Consequently, numerous studies have been conducted to find new alternative sources of antimicrobial agents, especially from plants. The aims of this project were to examine the antimicrobial properties of essential oils distilled from Australian-grown *Ocimum tenuiflorum* (Tulsi), to quantify the volatile components present in flower spikes, leaves and the essential oil, and to investigate the compounds responsible for any activity. Broth micro-dilution was used to determine the minimum inhibitory concentration (MIC) of Tulsi essential oil against selected microbial pathogens. The oils, at concentrations of 4.5 and 2.25% completely inhibited the growth of *Staphylococcus aureus* (including MRSA) and *Escherichia coli*, while the same concentrations only partly inhibited the growth of *Pseudomonas aeruginosa*. Of 54 compounds identified in Tulsi leaves, flower spikes, or essential oil, three are proposed to be responsible for this activity; camphor, eucalyptol and eugenol. Since *S. aureus* (including MRSA), *P. aeruginosa* and *E. coli* are major pathogens causing skin and soft tissue infections, Tulsi essential oil could be a valuable topical antimicrobial agent for management of skin infections caused by these organisms.

## Introduction

The use of medicinal plants in traditional medicine has been described in literature dating back several 1000 years (Chang et al., 2016). Books on Ayurvedic medicine, written in the Vedic period (3500–1600 B.C.) describe practices, including the use of medicinal plants, that formed the basis of all other medical sciences developed on the Indian subcontinent (Pattanayak et al., 2010). In modern complementary and alternative medical practice, plants are the primary source of therapeutics and each part of the plant, including the seeds, root, stem, leaves, and fruit, potentially contains bioactive components (Jiang et al., 2014, 2015; Mandave et al., 2014; Sun et al., 2014). The main bioactive components in medicinal plants are considered to be combinations of secondary metabolites (Singh et al., 2010; Wu et al., 2016). There are many advantages and benefits associated with the use of medicinal plants, the main ones being their cost-effectiveness and global availability. Their safety compared to other medicinal products and the lack of major side-effects are other clear advantages (Niu et al., 2011). However, plant metabolism is very variable and before medicinal plant extracts or products are approved for primary health care, they need to be standardized, subjected to stringent quality control and assessed to ensure their safety (Mantri et al., 2012; Olarte et al., 2013).

Among the medicinal plants, aromatic herbs are a rich source of biologically active compounds useful both in agriculture and medicine (Mathela, 1991; Cutler and Cutler, 1999). Of these, *Ocimum tenuiflorum*, also known as *Ocimum sanctum*, Tulsi, or Holy Basil from the family Lamiaceae has been described as the “Queen of plants” and the “mother medicine of nature” due to its perceived medicinal qualities (Singh et al., 2010). It has been one of the most valued and holistic herbs used over years in traditional medicine in India and almost every part of the plant has been found to possess therapeutic properties (Singh et al., 2010). Traditionally, Tulsi is used in different forms; aqueous extracts from the leaves (fresh or dried as powder) are used in herbal teas or mixed with other herbs or honey to enhance the medicinal value. Traditional uses of Tulsi aqueous extracts include the treatment of different types of poisoning, stomach-ache, common colds, headaches, malaria, inflammation, and heart disease (Pattanayak et al., 2010). Oils extracted from the leaves and inflorescence of Tulsi have been claimed to have numerous useful properties, including as expectorants, analgesics, anti-emetics, and antipyretics; stress reducers and inflammation relievers; and as anti-asthmatic, hypoglycemic, hepatoprotective, hypotensive, hypolipidemic, and immunomodulatory agents (Singh et al., 2010).

Several scientists have examined pharmacological effects of Tulsi products obtained by different extraction methods, such as steam distillation, benzene extraction and petroleum extraction. Prakash and Gupta (2005), reviewed all the scientific studies of the therapeutic significance of Tulsi and eugenol, a major component of Tulsi. These pharmacological studies may be helpful to establish a scientific basis for the therapeutic use of this plant, especially in regard to the pharmacological effect on the central nervous system, immune system, cardiovascular system, reproductive system, and the gastric and urinary systems.

Skin and soft tissue infections (SSTIs) are the cause considerable morbidity and cost to the community. Major causes of these infections are *Staphylococcus aureus*, *Pseudomonas aeruginosa*, and *Escherichia coli* and SENTRY Antimicrobial Surveillance Program, 2009 (Dryden, 2009, 2010). Although infections are often mild or moderate in severity, severe cases may require hospitalization and treatment with oral or parenteral antimicrobial agents. For instance, in 1995 more than 43,000 patients required hospitalized for treatment of SSTI in Scotland and 300,000 in the US (Eron et al., 2003). In recent years, SSTIs have become more difficult to manage due to the increasing occurrence of multidrug-resistant pathogens. To avoid the expansion of multidrug-resistant pathogens clinically, it is essential to differentiate between SSTI which require antibiotic treatment and those that do not. A recent survey in Europe reported that a major percentage of physicians prescribe systemic antibiotics for the treatment of conditions, such as MRSA-colonized ulcers or broken skin surfaces, that do require systemic antibiotics (Dryden, 2010). Essential oil or its components may be valuable agents for the treatment of mild or moderate skin infections or colonized ulcers, preventing progression to more serious infections and minimizing the unnecessary antibiotic use and the associated development of resistance.

The aims of this study were to (i) examine the antimicrobial properties of Tulsi essential oil, (ii) analyze the volatile composition of leaves, flower spikes, and extracted oil from Tulsi plants grown in Australia using headspace–solid phase microextraction–gas chromatography–mass spectrometry (HS-SPME-GC-MS), and (iii) after reviewing the literature, suggest which volatile compounds are most likely to be responsible for the antimicrobial activity of Tulsi oil. To the best of our knowledge this is the first analysis the Australian-grown fresh Tulsi flowers spikes, leaves, and the essential oil extracted from flowers and leaves using HS-SPME-GC-MS.

## Materials and Methods

### Source of Tulsi

Tulsi (*Ocimum tenuiflorum*), Voucher number PHARM-14-0028 obtained from the Medicinal Plant Herbarium at Southern Cross University, NSW, Australia, was used in this experiment. Fresh leaves and inflorescence (350 gm) were steam-distilled for 6 h in an essential oil Steam Distiller (Modified Clevenger apparatus) (Steam Distillation Apparatus, Crucible, Sacramento, CA, USA). The yield of volatile oil (weight of oil/weight of leaves made into a percentage) obtained was 0.57% v/w. The yellow colored volatile oil was stored in a sealed container at <4°C in the dark until needed.

### Antimicrobial Activity of Tulsi Essential Oil

The bacterial strains used in this study were *S. aureus* ATCC 25923, clinical isolate of methicillin-resistant *S. aureus* (MRSA) NCTC 6571 “Oxford Strain”, *E. coli* ATCC 25922 and *P. aeruginosa* ATCC 27853.

The extracted oil was emulsified in Mueller–Hinton Broth (MHB, Oxoid, Adelaide, SA, Australia) by the following method: 90 μl of the essential oil and 10 μl of DMSO were added to a sterile Eppendorf tube (Sarstedt, Technology Park, SA, Australia). The solution was mixed by vortexing then 900 μl of the MHB was added in 30 μL aliquots, with brief vortexing between each addition. The broth dilution method was used to determine the minimal inhibitory concentration (MIC) of the Tulsi essential oil for each bacterial species (Wiegand et al., 2008). Two-fold dilutions of essential oil, diluted, and solubilized as described above, beginning at 9% (undiluted), in volumes of 50 μl were prepared in MHB in a 96-well sterile flat bottomed microtiter plate (Corning, Hickory, USA), then 50 μl of bacterial suspension was added to each well, such that the final concentration was 5 × 10^5^ cfu in each well. The oil mixture was further diluted in the test (1:2) by the bacterial suspension, resulting in a solution containing of 4.5% essential oil in the first well. Plates were incubated for 24 h at 37°C in the dark on an orbital shaker at 100 rpm to prevent adherence and clumping. After incubation, the optical density of the contents of each well was determined using a spectrophotometer at 620 nm (Omega BMG LabTech, Ortenberg, Germany). For the minimal bactericidal concentration (MBC), 100 μl aliquots from each well were plated onto MHB agar and viable counts were determined after incubation for 24 h at 37°C (Clinical and Laboratory Standards Institute, 2012).

IBM SPSS (Statistical Package for the Social Sciences) (v.22, IBM Corporation, New York, NY, USA) was used for the statistical analysis of the amount of bacterial growth when treated with different concentrations of Tulsi essential oil. An alpha level of 0.05 was assumed for the determination of statistical significance. Analysis of variance (ANOVA) was followed by *post hoc* Tukey test to compare the amount of bacterial growth in wells containing different concentrations of essential oil. Data was calculated from two different experiments each conducted in triplicate.

### Isolation and Identification of Volatile Compounds from Leaves, Flower Spikes, and Oil

Fresh leaves, and flower spikes of Tulsi *Ocimum tenuiflorum* from the same source as the essential oil (distilled as described above) were collected from the Chinese medicinal garden at RMIT University, Bundoora Campus (Melbourne, VIC, Australia) in the summer of 2012; the temperature range during the growing season was 22–35°C. The samples were kept on ice after collection and during transportation to the laboratory, where 0.15 g of the inflorescence, fresh leaf material ground in a mortar and pestle unit, or essential oil was placed in a 4-ml clear, screw-top vial and sealed with a black polypropylene open-top cap and a PTFE (polytetrafluoroethylene)/silicone septum (Agilent Technologies, Santa Clara, CA, USA) and used immediately.

### Extraction of Volatile Compounds by HS-SPME

Extraction of the volatile compounds from the ground leaf material, flower spikes and oil was performed by headspace–solid phase micro-extraction (HS–SPME) using modified protocol of Yamani et al. (2014). A 85-mm polyacrylate (PA) fiber fitted to a manual sampling fiber holder (Supelco, Bellefonte, PA, USA) was conditioned according to the manufacturer’s instructions by placing into the gas chromatograph (GC) injection port at 250°C for 30 min before use. The preconditioned PA fiber was allowed to cool then inserted into the headspace of the vial containing the sample, and then the whole system was placed in a heating block at 40°C for 50 min. The volatiles were then desorbed by placing the fiber in the GC injection port for 5 min. The equilibrium time profile was developed using the method of Da Porto and Decorti (2008) with slight modifications; vials were placed in the heating block at 40°C instead of 30°C in order to extract all the compounds that might be present under hot conditions on summer days in Melbourne. Moreover, heating at 40°C resulted in an increased amount of volatile compounds on the fiber and a higher number of resulting peaks compared to heating at 30°C.

### Gas Chromatography–Mass Spectrometry

Identification of the volatile compounds from leaves, flower spikes and oil was performed using an Agilent 5973 GC hyphenated mass spectrometer (MS) system fitted with a DB-5 MS (5%-phenyl)-methylpolysiloxane fused silica column with dimensions 30 m × 250 μm, i.e., film thickness 0.25 μm, (Agilent Technologies, Mulgrave, VIC, Australia). Helium (99.99% purity) was used as the carrier gas at a flow rate of 1.5 ml/min. The split ratio was 50:1. The initial GC oven temperature was 40°C for 3 min, after which it was raised from 40 to 250°C at 6°C/min, where it was held for 5 min. The injection port, transfer line, and source temperatures were 250, 280, and 230°C, respectively. The mass scan range was 41–415 m/z. Data acquisition and processing were performed using MSD ChemStation (E02.00.493) (Agilent Technologies, Mulgrave, VIC, Australia). Qualitative identification was performed using GC–MS reference libraries (Adams 2007, Wiley 7th edition, and NIST 2.0) using a 80% similarity match cut off value. Concentrations of the studied compounds were calculated from the peak areas in the total ion chromatograms. The relative abundance of each compound was obtained from electronic integration measurements using the mean of three replicates. Kovats standard retention indices were determined from the retention times of a series of *n*-alkane mixture analyzed under identical conditions.

### Cytotoxicity Testing

The cytotoxicity of the tulsi extract toward HepG2 human cells was measured using Presto-blue^®^ Cell Viability Reagent kit (number A13262). HepG2 cells were seeded at 4 × 10^4^/well on a 96-well plate in 100 μL of growth medium and incubated for 24 h at 37°C. The medium was then removed, and the cells were washed with phosphate buffered saline (PBS). Samples were diluted in treatment medium (WME supplemented with 2 mM l-glutamine and 10 mM HEPES) and 100 μL of the samples were applied to the cells, then the plates were incubated at 37°C for 1 h. The treatment medium was removed, and the cells were washed with PBS. PrestoBlue reagent was directly added to cells in culture medium (96-well plate 90 μL 10 μL) and cells were incubated for 1–2 h at 37°C. The absorbance at 570 nm was determined using spectrophotometer (Omega BMG LabTech, Ortenberg, Germany).

## Results

### The Antimicrobial Activity of Tulsi Essential Oil

**FIGURE 1:**
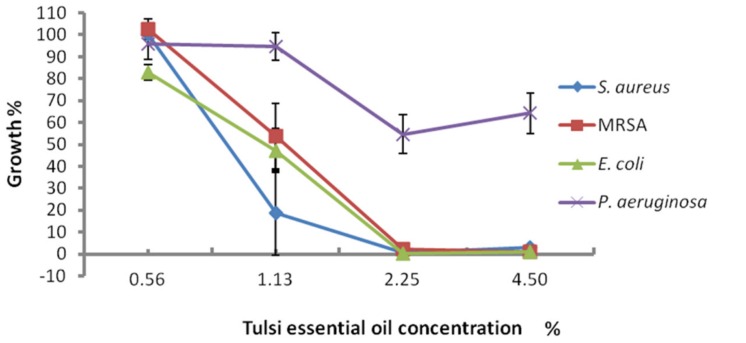
**The antimicrobial effect of different concentrations of Tulsi essential oil on the growth rate of four bacterial strains**.

Tulsi oil at concentrations of 4.5 and 2.25% completely inhibited the growth of *S. aureus*, including MRSA and *E. coli*, while the same concentrations only partly inhibited the growth of *P. aeruginosa* (**Figure 1**). The MBC results showed that Tulsi essential oil had only bacteriostatic activity against the examined bacterial strains. Viable bacterial counts were not measured because the plates were confluent indicating bacteriostatic activity. Statistical analysis of the spectrophotometric results showed that both the concentration of Tulsi oil and bacterial species used significantly affected the amount of growth (*P* < 0.05) (**Table 1**). The percentage of bacterial growth was lower overall when bacteria were treated with Tulsi oil at a concentration of 4.50% (17.29) and 2.23% (15.07) in comparison to 1.13% concentration (56.62). The main effect on the bacterial species growth for all essential oil concentrations was significant as well. The percentage of bacterial growth was lower for *S. aureus* (13.03), MRSA (18.58), and *E. coli* (17.99) than for *P. aeruginosa* (68.78). Overall, *P. aeruginosa* showed higher resistance to the antibacterial treatment with Tulsi oil, compared to three other bacteria used in the test. On the other hand, the mean difference of the percentage of bacterial growth between *S. aureus*, MRSA and *E. coli* was not significant *p* > 0.05.

**Table 1:** Analysis of variance (ANOVA) main effect of independent variables: tests of between-subjects effects.

Bacterial growth (%) (Dependent variable)	Tests of Between-Subjects Effects	
Independent variable	*F*	Significance
Concentration	28.300	0.000
Bacteria	29.502	0.000
Replicate	2.423	0.126

### Tulsi Volatile Composition

The analysis of the Tulsi leaves, inflorescence and essential oil by HS-SPME and GC/MS resulted in the identification of 54 volatile components (**Table 2**). The most abundant of the volatile components were monoterpenes and sesquiterpenes; in particular monoterpenes such as camphor, cineole, estragol, and eugenol, followed by sesquiterpenes, such as germacrene, caryophyllene, bisabolene. Nineteen major compounds were present in all samples from all parts of the plant, but at different concentrations. Overall, there were no major differences between the presence of the most prevalent volatile compounds identified from the leaves, inflorescence, and the essential oil, but minor compounds were frequently identified in only one or more sample types. The most common compound detected was camphor, with slightly different concentrations in the essential oil (31.5%), leaves (24.2%), and inflorescence (22.6%). This was followed by eucalyptol with higher concentration in both essential oil and leaves (18.9 and 13.47%, respectively) than in the inflorescence (1.2%). The third most commonly identified compound was eugenol that comprised 23.7% of the total volatile compounds in the leaves, 13.8% in the essential oil and 7.5% in inflorescence.

**Table 2:** Volatile compounds extracted from flower spikes, flower with nectar, and leaves of Tulsi using HS-SPME/GC-MS.

No	Compound (Adams KI)	Class	LRI	PtR	RT	Percentage in flower spikes	Percentage in leaves	Percentage in essential oil


1	Methyl isovalerate	E	766	5.42	5.31	0.1		0.02


2	Ethyl isovalerate	E	858	7.48	7.29	0.21		0.62


3	Tricyclene	M	926	9.16	9.18			0.05


4	Thujene	M	931	9.30	9.31			0.02


5	Alpha pinene	M	939	9.53	9.51	0.1	0.22	0.7


6	Camphene	M	953	9.93	9.98	0.52	0.34	1.71


7	Sabinene	M	976	10.59	10.63		0.14	0.17


8	Beta pinene	M	980	10.70	10.76	0.05	0.61	1.42


9	Octen 3 ol	M	979	10.67	10.76	0.25	0.18	


10	Myrcene	M	991	11.01	11.10	0.14	0.32	0.52


11	Phellandrene	M	1005	11.4	11.57	0.03		0.09


12	Terpinene	M	1018	11.75	11.87	0.1		0.18


13	Cymene -ortho-	M	1026	11.97	12.09			0.18


14	Limonene	M	1031	12.10	12.23	1.19	1.36	2.15


15	Eucalyptol	M	1033	12.15	12.31	1.19	13.47	18.85


16	Ocimene	M	1050	12.61	12.67	9.3	1.17	7.12


17	Terpinene	M	1062	12.93	12.98	0.07		0.41


18	Sabinene hydrate	M	1068	13.09	13.21	0.46	0.4	0.14


19	Terpinolene	M	1088	13.63	13.63	0.21	0.17	


20	Sabinene hydrate-trans-	M	1097	13.87	13.70			0.34


21	Carene	M	1002	11.32	13.80			0.1


22	Fenchone	M	1087	13.60	13.97	0.13	0.1	


23	Linalool	M	1098	13.89	14.115	0.297	0.06	0.302


24	Camphor	M	1143	15.03	15.40	22.55	24.15	31.52


25	Camphene hydrate	M	1150	15.21	15.52	0.17	0.21	0.24


26	Terpineol-delta-	M	1166	15.61	15.79		0.68	


27	Isoborenol	M	1162	15.51	15.73			0.06


28	Borneol	M	1165	15.58	15.93	0.62	0.69	1


29	Terpinen-4-ol	M	1177	15.88	16.14	0.08	0.2	0.84


30	Terpineol	M	1189	16.18	16.50		3.05	0.61


31	Estragol	M	1195	16.33	16.56	2.89	9.6	4.23


32	Eugenol	M	1356	20.02	20.15	7.49	23.67	13.77


33	Copaene	S	1377	20.48	20.66	0.26	0.32	0.26


34	Zingiberene	S	1494	22.94	20.79	0.1		


35	Bourbonene	S	1388	20.73	20.85		0.1	0.07


36	Elemene	S	1391	20.79	20.93	0.33		0.13


37	Guaiene	S	1410	21.20	21.35	0.29		0.05


38	Beta caryophyllene	S	1419	21.38	21.62	4.91	1.52	1.21


39	Bergamotene	S	1435	21.71	21.84	2.76	0.72	0.37


40	Sesquiphellandrene	S	1524	23.53	22.00	0.69	0.17	0.08


41	Farnesene	S	1458	22.19	22.12	0.56	0.12	


42	Sesquisabinene	S	1460	22.23	22.19	0.19		


43	Humulene	S	1455	22.13	22.37	0.54	0.19	0.11


44	Bicyclogermacrene	S	1500	23.16	23.14		0.04	


45	Germacrene	S	1480	22.65	22.89	11.29	3.77	1.88


46	Longipinene	S	1401	21.01	23.02	0.11		


47	Bisabolene-z	S	1507	23.20	24.02	0.15		


48	Muurolene	S	1500	23.06	23.22			0.06


49	Beta bisabolene	S	1509	23.24	23.38	10.65	3.29	2.2


50	Cadinene	S	1524	23.53	23.61		0.06	0.16


51	Alpha bisabolene	S	1504	23.14	23.96	16.71	5.38	3.83


52	Cubebene	S	1388	20.73	23.23	0.11		


53	Amorphene	S	1512	23.30	23.56	0.07		


54	Caryophyllene oxide	S	1583	24.69	24.93			0.03

*Blank spaces indicate a zero reading. Results show the average of three replicates. LRI – Linear retention index; PtR – Predicted retention time; E – Ester, M – Monoterpenes, S – Sesquiterpenes*.

Similar to the present study, Medina-Holguín et al. (2007) reported that the volatile compounds from fresh plant material displayed different compositions from those of the essential oil extracted from the same plants. Therefore, it is informative to compare the proportions of the different volatile compounds in different parts of the plant. Monoterpenes, especially eugenol, and estragole were present in the highest amounts in the leaves (23.7 and 9.6%, respectively), while sesquiterpenes ocimene (9.30%), caryophyllene- β (4.9%), bergamotene (2.8%), germacrene (11.3%), beta bisabolene, (10.7%), and alpha bisabolene (16.7%) were more prevalent in the inflorescence. Conversely, some volatile compounds were absent from the oil and detected only in the leaves or inflorescence but in trace amounts (<1%) at each site.

### Cytotoxicity Test

**FIGURE 2:**
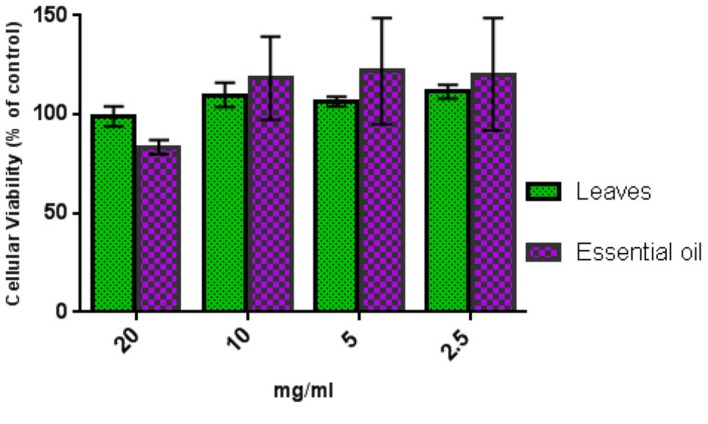
**Cell viability assay results of Tulsi essential oil and leaves extract**.

The cytotoxicity of the extracts decreased in relation to decreasing concentration. The cell viability decreased by less than 20% when the cells were treated with a concentration of 20 mg/ml. Both essential oil and concentrated leaf extract concentrations of 20 mg/ml were within the expected range as this concentration led to decreased cell viability by less than 20% and therefore were not considered cytotoxic. Concentrations below 20 mg/ml had no effect on the cell viability results, as shown in **Figure 2**.

## Discussion

### The Antimicrobial Activity of Tulsi Essential Oil

The agar diffusion method (well or paper disk) and the dilution method (liquid broth or agar) are the two basic techniques used for the examination of the antimicrobial activity of the essential oils. Of these, the agar diffusion method was commonly used in earlier studies. This method requires only a small quantity of the essential oil and is easy to implement. However, it is considered inappropriate for the essential oil examination for two reasons. Firstly, essential oil is comprised of volatile compounds that are likely to evaporate from disks during the incubation time. Moreover, due to the low solubility of essential oils in agar, volatile compounds may not diffuse well. Recently, the broth dilution method using 96 well flat-bottom microtitre plates has become the method of choice for determining the antimicrobial activity of essential oils. The activity of the essential oil is expressed as MIC and MBC. Kalemba and Kunicka (2003) strongly recommended that only MIC and MBC values should be published to facilitate comparisons between the results of different studies.

Our results show bacteriostatic activity of Tulsi oil at levels of 2.25–2.5 μg/ml against *S. aureus*, including MRSA and *E. coli*, but less activity against *P. aeruginosa* (**Table 3**; **Figure 1**). These results are broadly similar to those of studies that used disk diffusion or optical density reduction methods; however, there are differences in reported activity toward Gram positive and Gram negative bacteria (Mahmood et al., 2008; Helen et al., 2011; Mishra and Mishra, 2011) (**Table 3**). Mahmood et al. (2008), using a disk diffusion assay obtained zones of inhibition around both Gram-positive and Gram-negative species, but activity was better against *S. aureus* than against Gram-negative species. Mishra and Mishra (2011) reported good inhibition of both Gram-positive and Gram-negative species, indicated by a reduction in optical density; however, *P. aeruginosa* showed slightly better activity under these test conditions than *S. aureus*. Interestingly, Tulsi essential oil from one subspecies of *O. tenuiflorum* was more effective against *P. vulgaris*, *S. aureus, P. aeruginosa*, and *E. coli* than extracts from two other Tulsi subspecies (Helen et al., 2011). These reported differences in the antimicrobial activity of Tulsi oil could relate to the specific composition of the volatile compounds in the oil, determined by the geographical source of the plants or specific cultivar. Other reasons for the differing results could be methodology used for determining antimicrobial activity. It is also worth noting that *P. aeruginosa* is well known for both intrinsic and acquired resistance to many classes of antimicrobial agents (Poole, 2011). Studies using other essential oils also reported variable results, depending on the oil and analytical method selected. Prabuseenivasan et al. (2006), using the agar dilution method demonstrated variable antibacterial activity of 21 essential oils against a range of Gram-positive and Gram-negative bacteria. Several investigators reported that Gram-positive bacteria are more sensitive to essential oils in general than Gram-negative bacteria (Burt, 2004). For example, *S. aureus* was suggested to be inhibited by using the synergies of oregano and rosemary essential oils or carvacrol and 1,8-cineole (Honório et al., 2015).

**Table 3:** Antibacterial activity of *Ocimum sanctum* (Tulsi) essential oil.

Antibacterial Method	Species of bacteria	Concentration of oil (%) examined	MIC μg/ml	size mm	OD reduction	Reference
Disk-diffusion	*E. coli*	100		15.4		Mahmood et al., 2008
	*P. aeruginosa*	100		17.8		
	*S. aureus*	100		41.5		
Disk-diffusion	*E. coli*	100		12		Helen et al., 2011
	*P. aeruginosa S.*	100		12		
	*aureus*	100		12		
Optical density reduction	*E. coli*	10			0.40	Mishra and Mishra, 2011
	*P. aeruginosa*	10			0.71	
	*S. aureus*	10			0.62	
Broth micro-dilution method	*E. coli*	0.5–4.5	2.25			This study
	*P. aeruginosa*	0.5–4.5	>4.5			
	*S. aureus*	0.5–4.5	2.5			
	Methicillin-resistant *S. aureus* (MRSA)	0.5–4.5	2.25			

### Cytotoxicity Test

The effect of Tulsi extract and essential oil on cell viability was determined using the Presto–Blue test. The cells were treated with different concentrations of the extract, ranging from 2.5 to 20 mg/ml, and the results demonstrated that the cytotoxicity of the extracts decreased in relation to decreasing concentration. The cell viability decreased by less than 20% when the cells were treated with a concentration of 20 mg/ml. Both essential oil and concentrated leaf extract concentrations of 20 mg/ml were within the expected range as these concentrations led to decreased cell viability by less than 20% and therefore were not considered cytotoxic. Concentrations below 20 mg/ml had no effect on the cell viability.

### Comparison of Tulsi Volatile Compounds from Species Cultivated in Different Geographical Locations

Although the major types of volatile compounds identified by this study (monoterpenes and sesquiterpenes) were also identified in other studies, there are important quantitative differences in the distribution of these compounds in plants grown in other geographical areas. In previous reviews, Tulsi essential oil was generally reported to contain volatile compounds comprising monoterpenes such as linalool, estragol, eugenol, and small quantities of methyl cinnamate, cineole, tannins, camphor, and other compounds (Prakash and Gupta, 2005). However, the quantity of the volatile compounds identified and the major and minor compounds varied in different studies.

Also in agreement with the present study, an exhaustive survey by Singh et al. (2010) showed that extracts from fresh leaves and stems of Tulsi contain a number of sesquiterpenes and monoterpenes such as α-elemene, bornyl acetate, α- and β-pinenes, campesterol, and camphene, but the composition and amounts of the various compounds differed from those reported here. These differences could relate to the geographical origin of plant cultivars and environmental factors, which significantly influence the volatile composition and percentage, or to the method of extraction and analysis. **Table 4** displays examples of the major volatile compounds present in the essential oil extracted from Tulsi plants grown in different locations indicating that environmental factors strongly influence its chemical composition. Therefore, it was strongly suggested to analyze the composition of the volatile compounds of the essential oil before examining the oil for antimicrobial or other activity (Kalemba and Kunicka, 2003).

**Table 4:** Examples of the major volatile compounds present in the essential oil extracted from Tulsi plants grown in different location.

Geographical location	Source of essential oil	Major compounds	Concentration %	Reference
India	Leaves	Methyl eugenol	32.9	Helen et al., 2011
		cyclooctene	17.6	
India	Leaves	Eugenol,	27.4	Naquvi et al., 2012
		bornyl acetate	14.5	
		camphor	9.0	
India	Leaves, inflorescence, leaves and inflorescence	Methyl eugenol,	75.3, 65.2, 72.5	Kothari et al., 2005
		beta caryophyllene	6.4, 12.0,5.5	
Brazil	Leaves, inflorescence	Eugenol	79.0,17.6	Machado et al., 1999
		caryophyllene	9.8, 24.5	
Australia	Leaves	Methyl chavicol	87	Brophy et al., 1993
		camphor	4	
		beta caryophyllene	5	
Cuba	Leaves and inflorescence	Eugenol	34.3	Pino et al., 1998
		elemene	18.0	
		beta caryophyllene	23.1	
Germany	Leaves	Eugenol	38.2	Laakso et al., 1990
		methyl chavicol	14.4	
		eucalyptol	11.0	
		beta bisabolene	9.4	
		alpha bisabolene	7.5	
Australia (Victoria)	Leaves and inflorescence	Camphor	31.5	This study
		eucalyptol	18.9	
		eugenol	13.8	
		alpha bisabolene	3.8	
		beta bisabolene	2.2	
		beta caryophyllene	1.2	

### Bioactive Volatile Compounds Present in Tulsi

Camphor was the most abundant volatile compound present in essential oil (31.52%), leaves (24.15%), and flower spikes (22.55%) of the Australian-grown Tulsi (**Table 2**). Eucalyptol was the second most abundant volatile compound present in both essential oil and the leaves (18.85 and 13.47%, respectively). Camphor and eucalyptol are major components of the essential oils of three Greek *Achillea* species (*A. taygetea*, 26.6%; *A. holosericea*, 20.9%; *A. fraasii*, 16.3%). Furthermore, eucalyptol is the major constituent of the essential oil of *A. taygetea* and *A. fraasii* (25.7 and 11.9%, respectively), but was detected only in trace amounts (0.7%) in the oil of *A. holosericea*. The antimicrobial activity of these essential oils was assessed against six bacterial species; *S. aureus*, *S. epidermidis*, *E. coli*, *Enterobacter cloacae*, *Klebsiella pneumoniae*, and *P. aeruginosa* using the broth dilution technique. The oils of *A. taygetea* and *A. fraasii* showed strong to moderate activity against all six bacterial species, but the oil of *A. holosericea* was shown to have no antibacterial activity. In the same study, camphor was shown to be more effective than eucalyptol against the same bacterial species. It was proposed that the antibacterial properties of the essential oils of *A. taygetea* and *A. fraasii* are associated with their high content of camphor and eucalyptol (Magiatis et al., 2002). Both camphor and eucalyptol, identified as the abundant volatile compounds present in the essential oils of five taxa of *Sideritis* from Greece, were shown to possess some antimicrobial activity (Aligiannis et al., 2001). Moreover, camphor and eucalyptol standards obtained from Merck showed activity against *E. coli* and *S. aureus*, *Bacillus cereus*, *P. aeruginosa*, with camphor being more effective than eucalyptol (Mahboubi and Kazempour, 2009). Since both these compounds were identified as major components of Australian-grown Tulsi, we suggest that these substances may be responsible for the antimicrobial activity identified by this study.

The third most abundant compound identified in the present study, eugenol, formed 13.8, 23.7, and 7.5% of the total volatile compounds in the essential oil, leaves and inflorescence, respectively. Eugenol was also the main constituent of the Tulsi leaves grown in India, Brazil, Bangladesh, Cuba, and Germany (**Table 4**). In several reviews (Prakash and Gupta, 2005; Pattanayak et al., 2010; Singh et al., 2010) the therapeutic value of Tulsi leaves was attributed mainly to eugenol. Eugenol was identified as a major component (67%) of the essential oil of *Ocimum gratissimum*, and may be responsible for the antibacterial activity of that oil.

Another compound, β-caryophyllene, which comprised 4.9, 1.5, and 1.2% of volatile compounds in inflorescence, leaves and oil, respectively, of Australian-grown Tulsi, is a sesquiterpene which is widely distributed in essential oils of various plants. β- caryophyllene has been used for fragrance in cosmetics and as a food additive. β- caryophyllene also possesses antimicrobial activity (Alma et al., 2003; Legault and Pichette, 2007; Xiao-Yu et al., 2012). For example, Alma et al. (2003) examined the antimicrobial activities of essential oils extracted from the leaves of *Origanum syriacum*, which contained β- caryophyllene at a concentration of 12.6%. Using the agar-disk diffusion method, the oil was shown to have activity against *S. aureus*, *E. coli*, and *P. aeruginosa*. These findings suggest that β- caryophyllene may have contributed to the antimicrobial activity against the same three species of bacteria that was demonstrated in the present study.

Tulsi essential oil contains a valuable source of bioactive compounds such as camphor, eucalyptol, eugenol, alpha bisabolene, beta bisabolene, and beta caryophyllene. These compounds are proposed to be responsible for the antimicrobial properties of the leaf extracts. Further characterisation of individual compounds and their combinations will shed light on the most effective combination that can be used to treat skin infections. Such study could also include gene expression assays to determine the mechanism of action of these bioactive compounds. Once convincing evidence is obtained, formulations such as creams and gels could be derived from the Tulsi leaf extract, essential oil, or its bioactive compounds and tested for efficacy in treating skin infections. Considering Tulsi has been used for over 1000 years, such formulations are likely to be safer and readily accepted by consumers.

## Conclusion

In summary, the essential oil extracted from *Ocimum tenuiflorum* showed antimicrobial activity against *S. aureus* (including MRSA) and *E. coli*, but was less active against *P. aeruginosa*. Responses of *P. aeruginosa* to antimicrobial compounds was recently reviewed (Morita et al., 2014) and some compounds were found to interact with RND eﬄux pumps of the bacteria (Dreier and Ruggerone, 2015). In some other studies, *P. aeruginosa* has been shown to be more resistant than other most Gram negative bacteria to the action of the essential oils (Prabuseenivasan et al., 2006; Mahmood et al., 2008), but others have shown either increased sensitivity (Mishra and Mishra, 2011) or no difference in activity (Helen et al., 2011). A detailed analysis of the volatile compounds found in the essential oil and extracts from leaves and inflorescence revealed 54 different components that varied in presence and concentration in the three different sample types. A review of the literature suggested that the main components responsible for the antimicrobial activity of Tulsi oil were likely to be camphor, eucalyptol, and eugenol. β-caryophyllene may also have contributed to the antimicrobial activity of the oil but was present in smaller amounts. Since *S. aureus*, including MRSA, *P. aeruginosa*, and *E. coli* are major pathogens causing SSTIs, Tulsi essential oil could be a valuable topical antimicrobial agent for management of skin infections caused by these organisms or as a wound dressing to prevent infection. Early treatment or preventative measures may halt progression to more serious infection requiring systematic antibiotic therapy, and reduce the risk of development of resistance to valuable antibiotics.

## Author Contributions

EP and NM conceptualized the project. HY performed microbiological testing. EP, HY, NM, and MD analyzed the data and wrote the manuscript.

## Conflict of Interest Statement

The authors declare that the research was conducted in the absence of any commercial or financial relationships that could be construed as a potential conflict of interest.
